# How growth-induced stresses guide shape changes during animal morphogenesis: Mechanisms and implications

**DOI:** 10.1016/j.semcdb.2025.103661

**Published:** 2025-10-16

**Authors:** A. Erlich, S. Harmansa

**Affiliations:** a Université Grenoble Alpes, CNRS, LIPHY, Grenoble 38000, France; b Living Systems Institute, University of Exeter, Stocker Road, Exeter EX4 4QD, United Kingdom; c Department of Bioscience, University of Exeter, Stocker Road, Exeter EX4 4QD, United Kingdom

**Keywords:** Morphogenesis, Differential growth, Continuum mechanics, Residual stress, Tissue mechanics, Elasticity, Basement membrane

## Abstract

Morphogenesis, the process by which an organism develops its shape, is orchestrated by a complex interplay of genetic, biochemical, and mechanical factors. While myosin-driven contractility has been widely acknowledged as a critical driver of tissue shaping, emerging evidence suggests that differential growth (i.e. variations in growth rates within or between tissues) plays an equally vital role. Differential growth generates mechanical stresses that drive deformations at both cellular and tissue scales, shaping functional organ morphologies. This review introduces the core principles of growth mechanics in animal tissues and demonstrates how differential growth contributes to the generation of mechanical stresses that shape organs through processes such as folding, bending, and buckling, especially when different tissue layers or extracellular matrices impose external constraints. Furthermore, because cells can sense and respond to stresses, we highlight how integrating theoretical modelling with experimental data deepens our understanding of the feedback loops by which growth-induced stresses arise and mechanically guide functional shapes. Our aim is to engage developmental biologists by highlighting well-established insights from solid mechanics and plant biology on differential growth as a means to generate stress and shape tissue, complementing and extending the traditional focus on contractility.

## 1 Introduction

The remarkable diversity of shapes and patterns in living systems has captivated generations of biologists. Understanding the logic behind biological shape formation and its molecular regulation has long intrigued developmental biologists and, more recently, has drawn the attention of scientists in physics, materials science, and mathematics. Inspired by the vision D’Arcy Thompson described in his seminal work On Growth and Form [136], this interdisciplinary community started to investigate how morphogenesis is governed by the principles of physics and mechanics. The combination of powerful model systems, genetics, and in vivo and ex vivo biophysical assays, complemented by data-informed modelling frameworks, have advanced an integrated understanding of how growth-induced forces promote shape changes during animal development.

Morphogenesis - the process that shapes developing organisms - is tightly regulated by genetic and biochemical feedback-control [29]. In addition, morphogenetic reshaping of developing tissues is an intrinsically mechanical process that is fuelled by cellular force-generation [93,122,142]. Animal cells have the remarkable ability to generate forces in a controlled and active manner. Contractile forces originating from the cell’s actomyosin network drive dynamic deformations on the cell and tissue scale [49,90,112] and are well acknowledged in shaping animal tissues (discussed in several excellent reviews [51,62,92]). In contrast, the forces arising from growth-driven changes in tissue volume are often overlooked. These forces play an equally vital role in driving morphogenesis.

In this review, we attempt to bring together two areas that have largely developed in parallel but are highly complementary for studying morphogenesis: non-linear solid mechanics and developmental biology. Continuum mechanics, which describes how materials (including biological tissues) deform under forces and grow over time, has long been employed in fields such as engineering, biomechanics, and large-scale physiological modelling (e.g. the cardiovascular system [4,53,109,130, 127,131], tumour growth [9,13], wound healing [20,129], brain mechanics [55,146], and bone remodelling [31,40]). However, it is only in recent years that these methods have begun to significantly inform our understanding of morphogenesis, the developmental process by which a living tissue takes on its final shape. This review aims to fill that gap by highlighting how modelling growth and large-scale deformations using continuum mechanics can yield insights into developmental processes in animal tissues, as illustrated by examples such as sea urchin gastrulation [129], the formation of ribs in Ammonite seashells [41], and the intestinal crypt [6].

The mechanically rigid nature of plant cell walls makes growth-induced mechanical stresses and their anisotropy the primary drivers of tissue morphogenesis [64,75], being a fundamental concept for plant growth and morphogenesis. In contrast, animal cells lack rigid walls, allowing the contractile forces generated by actomyosin networks to deform and reshape animal tissues [2,79,92]. Much of our mechanistic understanding of tissue morphogenesis stems from studies of early embryonic development, when tissues are generally soft, deformable and mechanically permissive. In this context, actomyosin-driven contractility has emerged as a dominant force generator capable of driving large-scale tissue remodelling events, including epithelial folding, invagination, and convergent extension [85]. The mechanically permissive behaviour of early tissues facilitates contractility-driven deformations, enabling rapid and coordinated shape changes [21]. However, as development progresses, tissues undergo maturation, characterised by increased cellular organisation and tighter junctional integrity [106] and importantly, the deposition and remodelling of the extracellular matrix (ECM) [149]. These changes collectively lead to a progressive stiffening of developing tissues. In increasingly rigid tissues, the capacity of contractile forces to drive large-scale tissue deformation becomes increasingly constrained. Instead, growth-induced mechanical stresses, arising from differential growth, become more prominent drivers of morphogenesis. Growth-induced stresses accumulate over longer timescales and can induce shape changes even in the presence of significant mechanical resistance.

This review highlights developments in solid mechanics that can be of great utility in studying growth-induced stresses during animal morphogenesis. We examine how cell growth generates mechanical forces that drive shape changes from the cell to the organ scale. We summarise how growth-generated forces induce stresses that lead to deformations, thereby contributing to the emergence of complex morphologies. We begin by defining the concept of growth (Section 2) and then examine how growth can lead to stress accumulation during tissue expansion (Section 3). We highlight how data-informed modelling can enhance our understanding of how growth and the resulting stresses contribute to distinct morphological outcomes (Section 4). Building on these concepts, we discuss the role of growth-induced stresses in shaping both single-layer (Section 5) and multilayer tissues (Section 6). By integrating experimental and theoretical approaches, this review aims to provide a conceptual framework for understanding how growth-driven stresses contribute to animal morphogenesis.

## 2 Shaping tissues by growth-driven volume changes

**Fig. 1:**
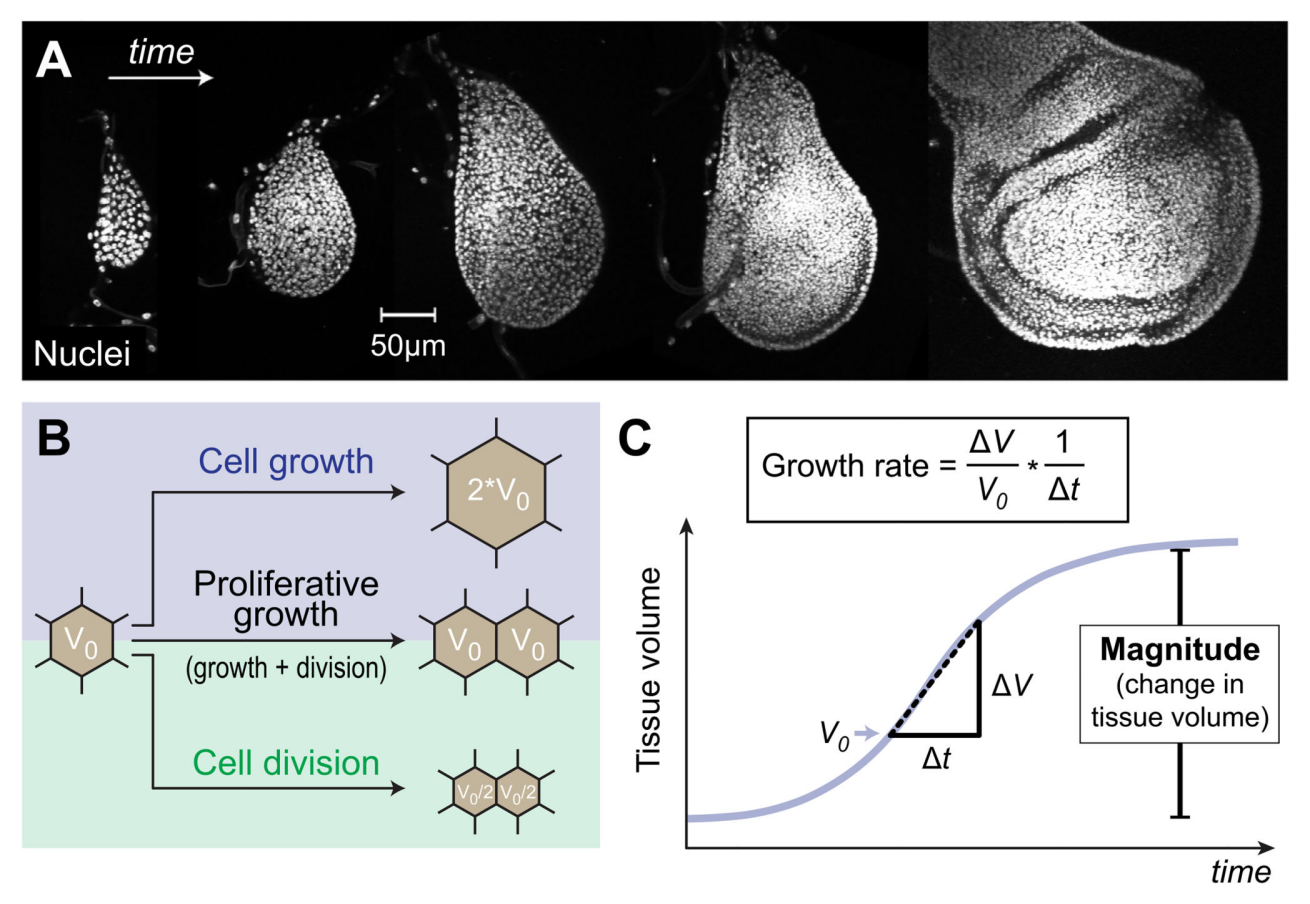
Developmental tissue expansion is driven by proliferative growth. (A) During the development of the *Drosophila* larval wing primordium, known as the wing disc, proliferative growth leads to an increase in cell numbers from a few hundred to 50,000 cells. This increase in cell number is associated with an increase in total tissue size and volume [19,59]. (B) Cellular growth is the process that leads to an increase in cell volume (*top*); such that the final volume is larger than the initial volume (*V*_0_). In contrast, the process of *cell division* (*bottom*) will divide the existing cellular volume in two (usually equally sized) daughter cells. Notably, in the absence of cell growth, division will lead to two daughter cells that are half the initial cell volume of the mother cell, i.e. *V*_0_/2. Proliferative growth (*middle*) is the combination of the two processes *cell growth* and *cell division*, representing the predominant mode of tissue expansion during animal development. Tissue expansion by proliferative growth leads to an increase in total tissue volume by increasing cell numbers while conserving the average cell volume (i.e. *V*_0_ in both daughter cells). (C) Example graph of a typical sigmoid growth curve (blue), illustrating the developmental change in tissue volume over time. The temporal changes in volume Δ*V* over a given period Δ*t* are described by the growth rate with unit [1/h]. Note, that growth rates are usually normalised to the initial tissue volume *V*_0_ to provide a standardised measure of growth that is independent of the system’s starting size. The total increase in tissue volume is referred to as the magnitude of growth.

Growth refers to an increase in size and mass of an organism or its parts such as tissues and organs. During animal development growth can produce drastic changes in tissue size, as observed during the growth phase of the *Drosophila* larval wing imaginal disc (see Fig. 1A). At the tissue level, growth manifests itself as a change in volume and mass which can have a directionality: If there is more growth in one direction than in another, growth is *anisotropic*. Growth at the tissue level, whether it is isotropic (i.e. equal in all directions) or anisotropic (i.e. directional), is a coarse-grained process and as such partially blind to what is happening at the cell scale. To explore growth on the cell scale, we need to distinguish two interconnected processes: *Cell growth* and *cell division* (see Fig. 1B). The former refers to a temporal increase in cell volume, i.e. when a cell gets bigger during the cell cycle. Physically, cell growth is a complex swelling phenomenon in which the cell takes advantage of osmosis and uses passive ion channels as well as active pumps to attract extracellular fluid in a carefully orchestrated process known as cell volume regulation [24]. Notably, *cell growth* encompasses the temporal increase in cellular mass (i.e. protein content) which is directly linked to the temporal increase in cytoplasmic, nuclear and organelle volumes. Cell growth by itself does not affect how cells are connected to each other, i.e. it does not alter the topology (i.e. connectivity) of the cell network. Conversely, *cell division* changes the topology of the cell network by adding an additional cell. This idealised distinction into cell growth and division is important because the swelling of a single cell or the division of a cell may be indistinguishable at the tissue level, creating a local volume increase with potentially some directionality (anisotropy). However, at the level of the cell network the two processes are dramatically different.

In reality, changes in cellular volume by growth and cell number by division rarely occur independently. In most tissues, cell growth is followed by cell division, i.e., the two modes occur concurrently in repeated cycles [24,52,87,135] known as *proliferative growth* (see Fig. 1B). The tight coordination of these two processes ensures that cell density and the average volume of cells within a given tissue and cell type are precisely controlled [24,52,87]. However, there are some notable exceptions in which the coordination between cell growth and division is weak. Notably, experiments in the *Drosophila* wing disc demonstrate that cell growth is dominant over cell division, as cells can continue to grow even when the cell cycle (i.e. division) is slowed down: Genetically reducing cell cycle rates results in wing discs that consist of fewer but larger cells while not affecting overall tissue size [97,98]. In the mammalian heart, organ size increases initially via proliferative growth during embryonic development but postnatally switches to hypertrophic growth, characterised by an increase in cardiomyocyte volume without further division [37]. Conversely, cell divisions without prior growth lead to a progressive reduction in cell volume with each new division (as shown in Fig. 1B, *bottom*). Such reductive divisions, where the cell cycle oscillates between DNA synthesis and mitosis (M-phases), are predominantly observed during early embryonic cleavage divisions in several metazoans [115]. Despite these exceptions, proliferative growth by tight coupling between cell growth and division remains the predominant mode during development.

Although cell proliferation rates can be readily quantified using various histological assays [116,117,152], the aforementioned exceptions to proliferative growth demonstrate that changes in cell number are not always a reliable proxy for tissue growth. As described, growth involves temporal changes in tissue mass and volume and is therefore ideally characterised as a dynamic volumetric process (Fig. 1C). Volumetric imaging techniques, such as two-photon and light sheet microscopy, enable the quantification of tissue volume changes during development [59]. The change in tissue volume (Δ*V*) over a period of time (Δ*t*) determines the growth rate of a given tissue (Fig. 1C). Normalisation to the initial tissue volume (*V*_0_) makes the growth rate independent of the tissue size and allows the comparison of growth dynamics between different tissues or time points. Such dynamic measurements provide valuable parameters to inform computational modelling frameworks, helping to investigate how growth-driven volume changes mechanically influence shape evolution (as outlined in Section 4).

Growth and shape generation are inherently linked processes during animal development. Numerous examples show that growth-driven tissue expansion is closely associated with major shape changes throughout morphogenesis. The spatial-temporal patterns of growth determine the shape of diverse structures, including the beaks of Darwin Finches [48,3], epithelial organs [59,139], appendages [147] and complex multilayered tissues [77,114,134].

Collectively, these examples support the modern view that ‘growth laws’, i.e. spatial-temporal control over growth dynamics, govern the shape of developing biological structures. To understand how growth drives shape, we must consider its mechanical role as a force generator and how interactions between growing materials and their surroundings lead to stresses (see Table 1 for definitions of physical terms) that induce elastic deformations at the cell and tissue scale.

## 3 The case for growth-induced mechanical stress

### 3.1 The multi-scale nature of morphogenesis

**Fig. 2:**
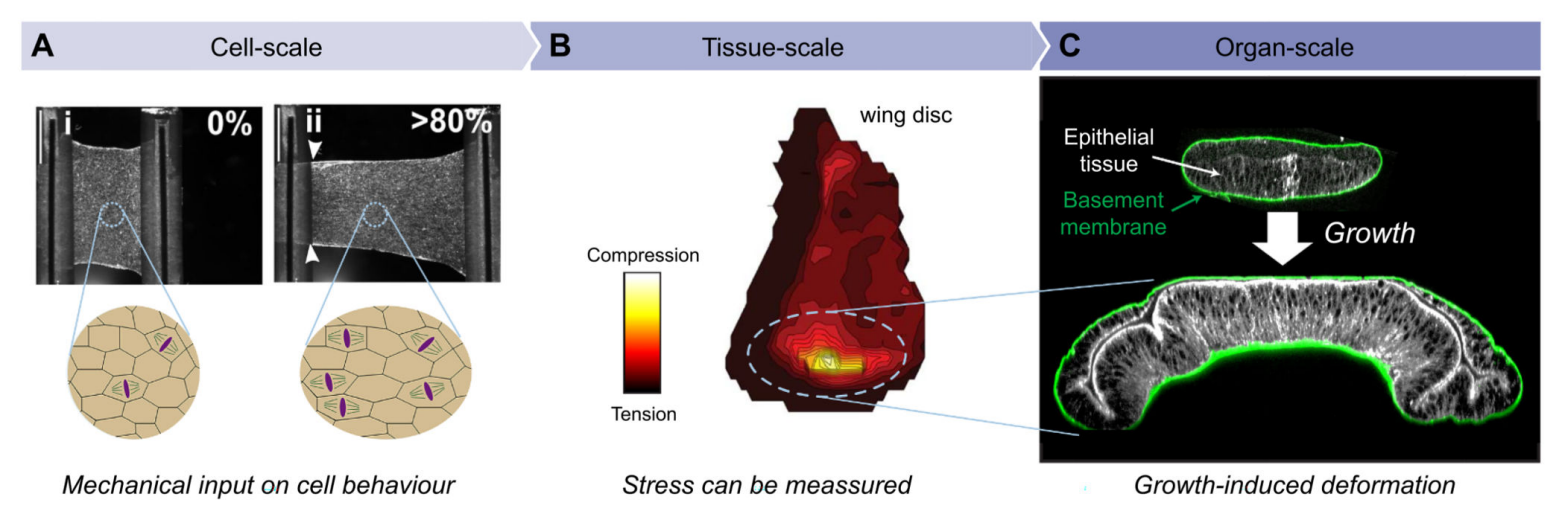
Morphogenesis as a multi-scale problem. (A) Stretching experiments of an epithelial tissue monolayer (quasistatic monotonic extension, creep tests, and cyclic loading). Tissue-level stretch causes a feedback that upregulates cell division oriented perpendicular to the strain, thus affecting the cell scale (images from Harris et al. [60] and Nestor-Bergmann et al. [96]).(B) Photo-elasticity measurements of the *Drosophila* wing disc show a tissue-level stress pattern, with central compression and peripheral tension of cells (images from Nienhaus et al. [99]). (C) Differential growth between multi-layered structures, here an epithelial tissue and its underlying Collagen IV-rich basement membrane (marked by Collagen IV::GFP), explains the bending of the *Drosophila* wing disc. Panel adapted from Ref. [59], licensed under CC BY 4.0.

During animal development, epithelial tissues serve as the primary structural units of growing organs. Composed of tightly interconnected cells, epithelia form sheets that undergo precisely coordinated shape changes sculpting them into complex functional morphologies. Understanding the tissue mechanics and stresses associated with epithelial growth during animal development poses challenges at all relevant levels: From the cell to the organ scale, revealing the multi-scale nature of morphogenesis (Fig. 2). Growing tissues are active materials and cells constantly interact with and respond to their mechanical environment. For example, cells sense and collectively respond to deformation, as demonstrated by stretching experiments of an epithelial monolayer of Canine Kidney (MDCK-II) cells (Fig. 2A) [60]. In the absence of deformation, cell division events are randomly oriented; however, they align with the principal axis of deformation when stretched [96]. Analogously, tissue stretching stimulates cell proliferation (i.e., growth), whereas compression inhibits growth [84] and even leads to cell death and extrusion [100]. These findings reveal how tissue-scale deformations evoke coordinated response patterns at the cell scale.

Although quantitative examples of stress patterns (and changes thereof) during development are rare, there are several techniques that can assess stress in epithelial tissues. Notably, these techniques can be categorised into two groups: invasive and non-invasive. Invasive techniques include the introduction of tissue cuts via laser ablation [39,91,121], the injection of micro-droplets [25,107] or bio-printed force sensors [88]. However, these techniques require direct physical interference with the sample, which can lead to artefacts or disturb normal development. Notably, laser ablations are used to introduce controlled cuts to structures at the subcellular and multicellular levels that are destructive, i.e., they interfere with the integrity of the sample and hence do not allow probing of the same sample at later stages of development. In contrast, non-invasive techniques do not directly interfere with or destroy the sample. Examples are force inference [72,74,78], photo-elasticity measurements [99] and Brillouin microscopy [18,108]. Both force inference [74] and photo-elasticity [99] measurements support the presence of a tissue-scale mechanical gradient in the *Drosophila* wing imaginal disc. The latter study relied on optical retardance which arises from stress-birefringence, where mechanical stress aligns molecules anisotropically, altering refractive indices and causing a measurable phase shift in transmitted light. Visualising the optical retardance pattern in the wing disc via photo-elasticity measurements showed that retardance is highest in the centre of the imaginal wing disc, implying that this region is subjected to the highest compression (Fig. 2B). Collectively, these investigations demonstrate that stress patterns can be quantitatively assessed during animal development.

Notably, the presence of such patterns of internal (residual) stresses poses two important questions: (1) What causes such stresses to accumulate in developing tissues? (2) Could these internal stresses lead to growth-induced elastic instabilities and thereby contribute to shape generation? Indeed, the aforementioned build-up of compressive stress in the *Drosophila* wing disc is due to its multi-layered nature. Differential, growth-driven expansion between the tissue layer and its underlying basement membrane layer (a sheet-like extracellular matrix) leads to stress accumulation, which is responsible for organ-scale bending leading to the typical dome shape of the wing disc (see Fig. 2C). This is only one of many examples that support the notion that growth-induced stresses at the tissue scale drive organ-scale deformations, actively shaping morphogenesis.

### 3.2 Residual stress

Stresses during development are often associated with cellular contractility, e.g., through myosin activity [2]. However, there is another important way to create stress: Differential growth (the non-uniform expansion of tissue) produces residual stress, i.e. internal stress remaining even after removing all external loads. Differential growth can lead to situations where there is no space for the cellular material that was newly added by growth. Consider a growing tissue where central cells grow faster than the surrounding peripheral cells. Since the faster-growing cells will expand more than their slower-growing neighbours, they will not fit anymore in the provided space, creating a geometric frustration in the tissue, known as *incompatibility*. In this context, the *incompatibility* is of a geometric nature and necessitates deformation at the cost of growth-induced stress: The faster-growing core pushes outward, stretching the surrounding slower-growing tissue, which in response leads to the compression of central cells (i.e., build-up of pressure).

**Fig. 3:**
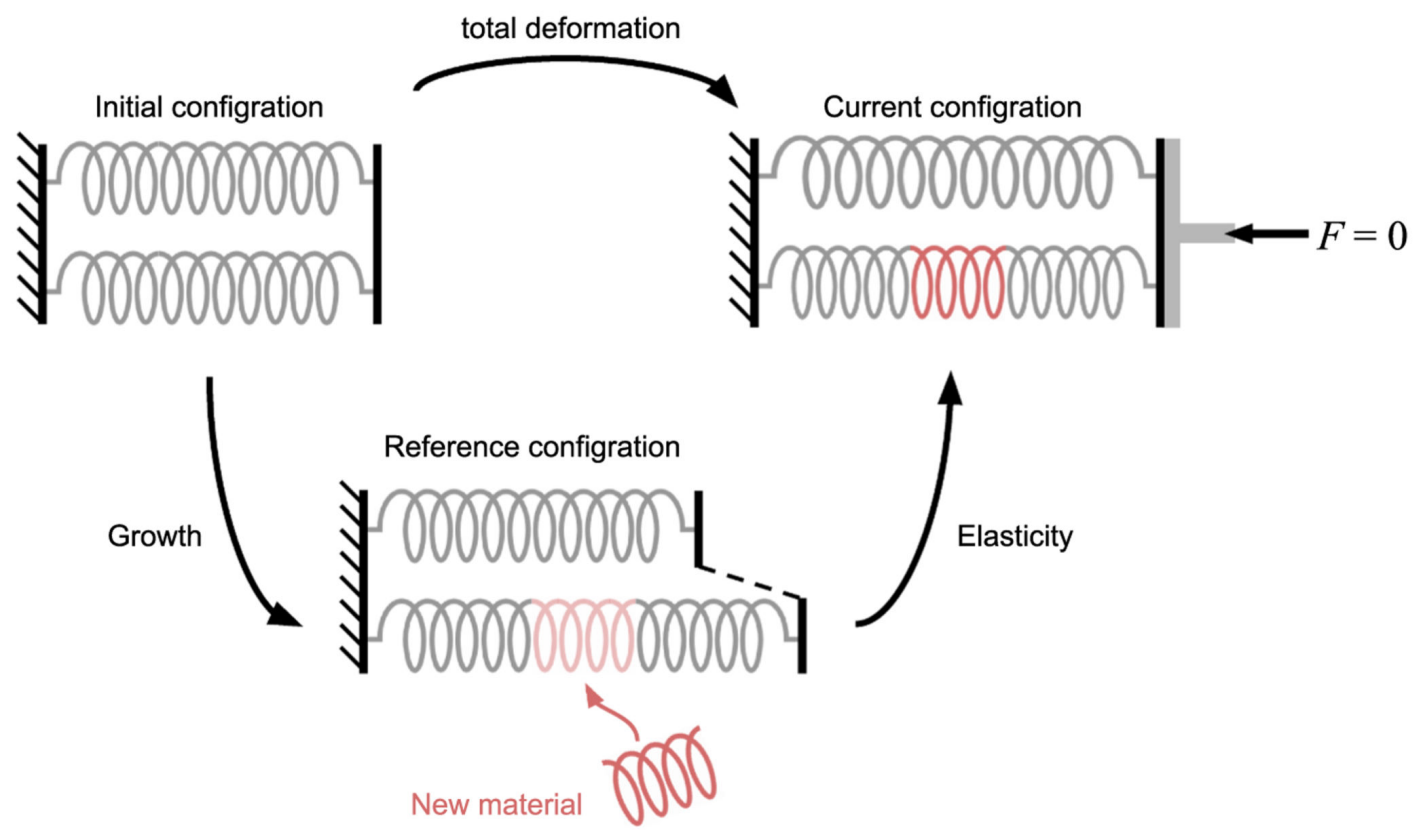
Illustration of residual stresses generated by differential growth in a minimal two-spring model. In the initial configuration both springs are of the same length and hence their geometry compatible (and *stress-free*). In the reference configuration, differential growth leads to geometric incompatibility as the rest length of only one spring (red) becomes longer than the other. This incompatibility is resolved in the current configuration by elasticity: the shorter spring is under tension and the longer spring under compression, resulting in a stressed equilibrium state even in the absence of an external force *F* (residual stress). The vertical wall is sketched as a piston to emphasise that walls cannot tilt in this thought experiment.

The build-up of growth-induced stress can be illustrated using a minimal example of two parallel springs (see Fig. 3). In the initial configuration (before growth occurs), we assume that both springs are identical and at rest, meaning that the actual spring lengths match their rest lengths, so that there are no forces in the springs and no elastic energy in the two-spring structure (they are both stress-free). Growth can be thought of as increasing the rest length of one spring. As new material is added to one spring (red in Fig. 3), the rest length of the grown spring becomes longer than that of the other spring that did not grow. In the stress-free reference configuration, the two springs of different lengths can no longer form a coherent structure within the original geometry marked by the vertical walls (*incompatibility*).

This apparent contradiction is resolved through elasticity. If the shorter spring is put in tension and the longer spring in compression, the current (i.e., actually observed) configuration has non-zero elastic energy, i.e., it is elastically stressed, even if no external force is applied (*F* = 0) (see Fig. 3 ‘current configuration’). In the above example, stress is essentially created by a growth mismatch between parts of a structure. If these parts were not connected into one coherent structure (Fig. 3, reference configuration: dashed lines), there would be no stress (the reference configuration is stress-free). But as the springs are connected into one coherent structure at the boundary (bold vertical lines in Fig. 3), differential growth leads to the accumulation of stress. Such elastic stress is referred to as residual stress, which is defined as the stress present in a structure in the absence of any external force [54,127].

Notably, the presence of residual stress has been demonstrated in many different animal tissues. It can be observed with the naked eye and becomes apparent when the integrity of the tissue is broken: introducing tissue-scale cuts leads to relaxation and tissue opening, indicating the relaxation of compressive growth-induced stress, as observed in arteries [27,56], the aorta [38,67,141], the heart [103,154], the trachea [58, 118], and the brain [23,150,153]. Residual stress is also observed in solid tumours [124], raising the question to which extent they are involved in controlling tumour growth [10,44]. Interestingly, recent work highlights the presence of dynamic residual stress patterns that guide organ growth and morphogenesis, such as in the chick gut tube [114] and optic cup [102], as well as in the *Drosophila* wing disc [45,59].

### 3.3 Morphogenesis – interconnected patterns of growth, stress and deformation

As we have seen in the previous section, differential growth can lead to a growth mismatch (*incompatibility*) which induces residual stress. This leads to a change in morphology, which in the previous 1D morphology is simply a change in the observable length of the network of springs. However, in 3D, a myriad of distinct growth patterns are possible (as we discuss in Section 5) that theoretically could induce a huge diversity of three-dimensional shapes and tune tissue- and organscale morphologies.

**Fig. 4:**
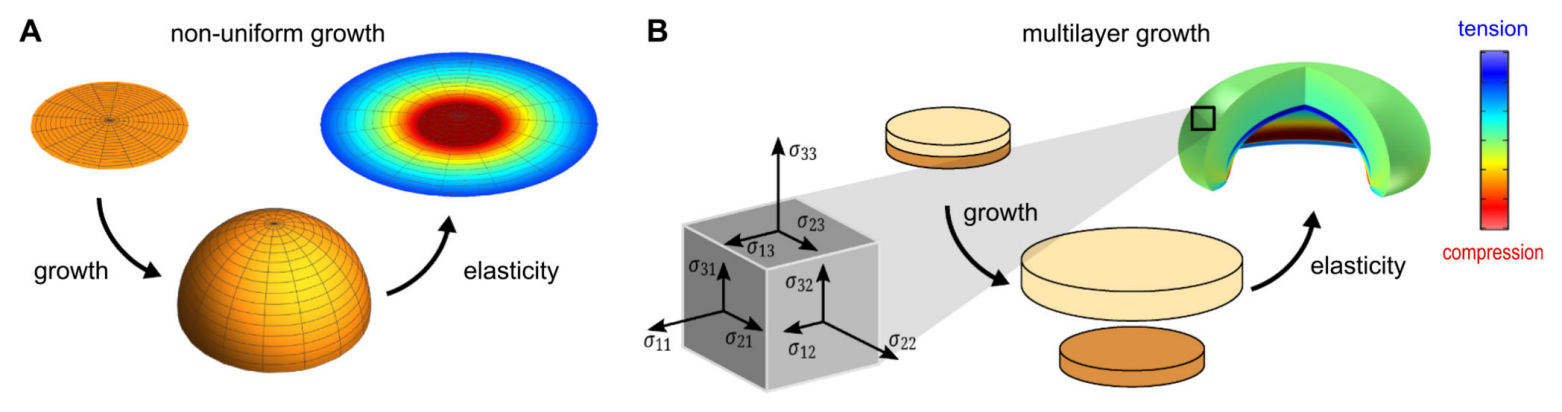
Non-uniform single layer growth versus uniform multi-layer growth. (A) Consider a flat 2D, unstressed disc (*top left*). An anisotropic non-uniform growth pattern (illustrated in Fig. 8 under “uniform *κ*_*G*_”) renders its relaxed configuration into a hemisphere embedded in the third dimension (middle). If the disc is confined to two dimensions (e.g., growth between glass plates), the 2D “flattening” will induce residual stresses (*top right*, stress levels are indicated by colour), even when the disc is free at its edges. (B) The complementary example: Two flat unstressed discs are connected as a sandwich structure (*top left*). As the classic stress cube shows normal and shear tractions acting on three perpendicular faces, mechanical stress in biological tissues is inherently a three-dimensional, tensorial quantity. While the growth pattern is perfectly homogeneous and isotropic in both individual layers, the top layer grows more than the bottom one (*middle* picture), thereby creating a geometric mismatch (i.e. the two discs have different surface areas and do not fit any more). Due to the geometric mismatch at the interface, elastic deformation is required to combine the two differently sized discs into one coherent object, which induces bending and stresses (*top right*). Panel adapted from Ref. [59], licensed under CC BY 4.0.

Let us consider the simple scenario of a single-layered and initially flat epithelial tissue and evaluate how differential growth can create complex morphologies. For simplicity, we consider this layer to be continuous, meaning without holes or internal interfaces and initially stress-free. We consider the growth of a 2D disc, which is initially planar and unstressed (Fig. 4A, *top left*). If the discs grew isotropically and homogeneously, i.e., the same way in all directions and the same way at the centre and boundary (and everywhere else), the disc would simply scale up and remain flat and stress-free (for reference, this particular scenario is illustrated in Fig. 7 “isotropic growth”). Conversely, if a planar unstressed disc is grown according to a non-uniform and/or anisotropic growth pattern, it grows incompatibly. Its hypothetical, stress-free configuration might escape into the third dimension in a hemispherical pattern (Fig. 4A, *middle*). Flattening the disc back to 2D would create a relatively complex stress pattern (colour coded in Fig. 4A). Even without flattening the disc, the incompatible growth pattern will in general lead to the accumulation of residual stress and hence stretch parts of the tissue. This would incur elastic energy, and the elastic equilibrium configuration would have a non-trivial shape and residual stress pattern. Therefore, even within a simple, single-layered tissue, an incompatible growth pattern will lead to stress accumulation resulting in deformations that change the morphology of the epithelial tissue.

A complex stress pattern (and hence a complex morphology) can also be created via comparatively simple growth patterns that change across interfaces. In Fig. 4B, we consider a stack of two discs, a bilayer. Let us assume that the two parts of the bilayer each grow isotropically (i.e., they expand uniformly in all directions) but at different rates (Fig. 4B, *middle*). Individually, the isotropic growth pattern in the layers would not create any residual stress by itself. But as the bilayer adheres across the disc-disc interface, and discs are therefore physically connected, the resulting stress profile (colour gradients) and morphology is complex.

The many possible growth patterns, and how they impact and deform tissues during morphogenesis, create a need to develop new theoretical tools and experimental protocols to deal with living and growing tissues. On the theoretical side, a major current limitation is a lack of a multi-scale theory of growth of epithelial tissues. The key issue is that growth is an inherently cell-based process. At the same time, cellbased models (such as vertex models) have the downside of being computationally costly and of producing tissue behaviour that is sometimes challenging to interpret through continuum mechanical theories. Therefore, a continuum theory that integrates micromechanical modelling is needed, so that we can take advantage of robust computational techniques like the Finite Element Method. In the following section, we will briefly review some theoretical tools that have been applied to growing tissues successfully. As we intend to focus in this review on the tissue scale, we will expand in more depth the continuum aspects to simulate tissue-scale mechanics.

## 4 Modelling frameworks to study the mechanics of growth

Understanding growth-induced residual stresses and their effects on shape and morphogenesis requires an appropriate description at the right level. This section focuses on providing such a description at the tissue level. During morphogenesis, living tissues can change shape and size very rapidly. Morphogenetic events include spectacular shape changes such as self-inversion [65], looping as in the case of the heart tube [110], or branching such as in lungs, kidney, and vascular networks [43]. As these examples illustrate, morphogenesis involves complex interactions between growth, non-linear solid mechanics, and changes in shape and size. The leading description at the continuum level is Morphoelasticity, a multiphysics framework coupling growth and non-linear elasticity [111].

### 4.1 Kinematics (geometry)

In traditional continuum mechanics, the single-layer tissue or multilayered structure is referred to as a “body”. It is common to consider the body in two different configurations: the *initial configuration* and the *current configuration*. The initial configuration ℬ_0_ describes the body in its initial state (*i.e*., before deformation) and is required to be *stress-free*. The current configuration ℬ_*t*_ describes the deformed body at time *t* and is usually stressed. Strain, a measure of the geometric deformation of a body ℬ_*t*_ with respect to the initial state ℬ_0_, is quantified through the deformation gradient **F** (note that we use here the terminology of elasticity in which *F* is a measure of *strains* and rotations and not force as in other areas of physics; see Table 1 for used mathematical symbols). The tensor **F** maps a material’s undeformed configuration to its deformed configuration by describing the local changes in position, orientation, and shape of material elements.

In morphoelasticity, we decompose the deformation gradient into a growth component, described by a growth tensor **F**_*g*_, and an elastic deformation component, described by the elastic deformation tensor **F**_*e*_, that is:

(1) $$F=F_{e}F_{g}.$$

**Fig. 5:**
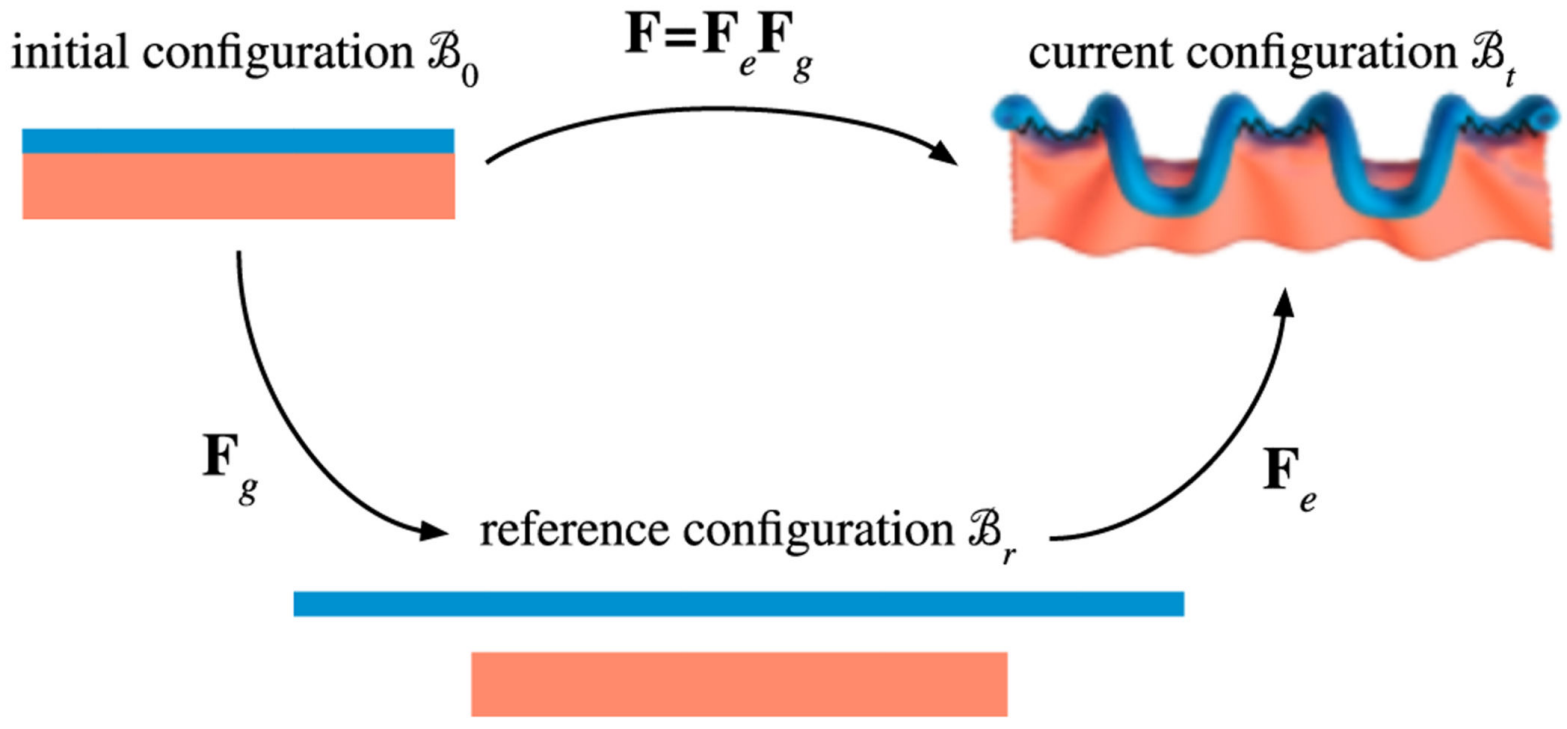
Kinematics of growth. The growth tensor **F**_*g*_ describes growth without stress, leading to an incompatible configuration. The elastic deformation gradient **F**_*e*_ restores compatibility by introducing residual stress in the current configuration. Adapted from Ref. [114].

The morphoelastic decomposition is illustrated in Fig. 5. Physically, **F**_*g*_ describes growth without stress, leading in general to an incompatible configuration of the body, the *reference configuration* ℬ_*r*_.

*Differential growth* means that parts of the body grow differently from each other; for example, one part of the body expands more than another or expands in a different direction. In the example of a bi-layer body (see Fig. 5), the blue layer expands more than the red layer (ℬ_*r*_ is *incompatible* [111,140]). We therefore need to introduce elastic deformation, described by **F**_*e*_, to resolve the geometric incompatibility: by stretching the smaller red layer and compressing the larger blue layer, we can obtain two compatible parts that can form a coherent structure. To minimise its elastic energy, the body will deform in 3D (if allowed), balancing opposing internal stresses in a stable equilibrium state, the current configuration ℬ_*t*_. For this reason, ℬ_*t*_ is no longer stress-free, even in the absence of any external loads or forces. This internal stress that persists even in the absence of loads is called *residual stress*.

### 4.2 Constitutive modelling of tissue mechanics

To understand how growth affects the mechanics of tissues, we need to relate the stresses within the tissue to the deformations it undergoes. Constitutive laws describe how a certain strain (resulting from deformation) of a material leads to a certain stress, i.e. they link strain with stress. A commonly used constitutive law for embryonic tissue is the neo-Hookean material. It extends linear elasticity to scenarios involving large deformations, nonlinear responses, and takes into account the material property of incompressibility. The latter means that elastic deformations locally do not change the tissue volume (bulk compression is prohibited) while other deformations such as shear, bending or torsion remain possible. The definition of incompressibility is that elastic deformations are volume-preserving (det **F**_*e*_ = 1). The neo-Hookean incompressible model is often used as a first approximation for biological tissues when large deformations occur and the tissue is also dominated by a high-water content, rendering it approximately incompressible. The stress-strain relationship, i.e. the constitutive law for the neo-Hookean incompressible model, is:

(2) $$\sigma =\mu \;F_{e}F_{e}^{T}-pI$$

where ***σ*** is the Cauchy stress tensor, the term *p***I** ensures that hydrostatic pressure (*p*) is applied isotropically, and *μ* is the shear modulus, characterising the material response to folding or stretching (see also Table 1). In the literature, for linear elastic materials, often the Young’s modulus *E* and Poisson’s ratio *ν* are employed. In the present case of incompressibility, *ν* = 1*/*2, and in this case one can relate the shear modulus ***μ*** to Young’s modulus by ***μ*** = *E/*3. Importantly, at high values of ***μ*** = (or *E*), the tissue is stiff against shear deformations.

To model the mechanical equilibrium, i.e. a generalised Newtonian force balance, we first make some assumptions relevant to biological tissues and the particular focus on residual (internal) stresses. As the timescale of growth is typically slow compared to elastodynamics (wave propagation in elastic media), we neglect all inertial effects. Further, we neglect body forces (such as gravity), and we assume that no traction forces are applied at the boundary *∂*ℬ_*t*_, meaning that we neglect, for instance, any externally applied hydrostatic pressure. Thus, the force balance reads:

(3) $$\nabla \cdot \sigma =0\;\text{on}\;{ℬ}_{t},\sigma n=0\;\text{on}\;\partial {ℬ}_{t}.$$

**Fig. 6:**
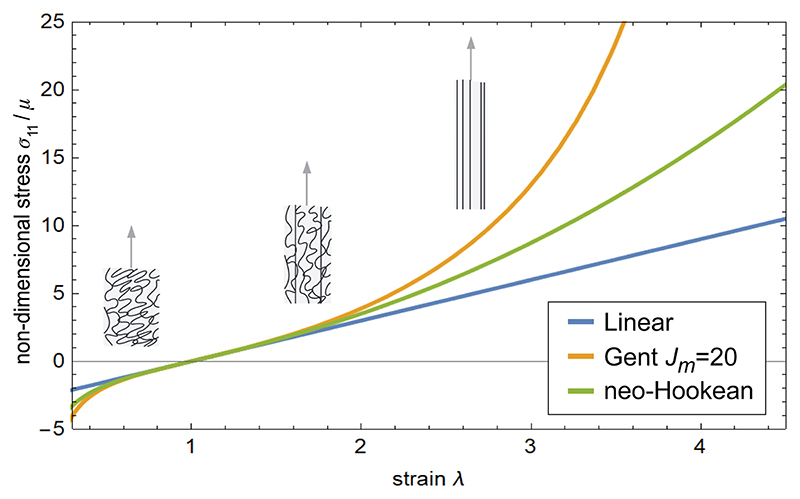
Stress-strain response of different models in uniaxial extension. The incompressible Gent model responds similarly to the incompressible neo-Hookean model at small strains (*λ* ≈ 1) but strongly rigidifies the material at larger strains (here *λ* ≈ 3). The insets show the microstructural explanation, in which inextensible fibres in the material get increasingly taut at large strains (insets from [66]). A linear (Hookean) force response curve is also shown.

The first equation states that inside the tissue region ℬ_*t*_ the internal stress tensor ***σ*** balances everywhere, so no net force accumulates. At the boundary *∂*ℬ_*t*_, ***σ*n = 0** says the stress on each surface element in the direction of the outward-pointing unit normal vector **n** vanishes, i.e., the surface is traction-free. Notably, in cases where more information about a tissue is available and where tissue behaviours are more complex, models other than the incompressible neo-Hookean model (Eq. (2)) will be necessary. For instance, a feature common to some biological tissues is *strain stiffening*, meaning that at large deformations, the tissue rigidifies. This is often due to the organisation of cross-linked polymer networks inside the tissue. If the tissue is stretched a little, the crumpled polymer chains respond as springs, similarly to an elastic Hookean response. As the tissue is stretched further, the chains gradually uncrumple and become taut. At that point, the inextensible taut chains provide a strengthened elastic response. A simple model capturing such a strain-stiffening effect is the Gent model [68]. Its key parameter *J*_*m*_ reflects the maximum amount of stretch (extensibility) the material can undergo before the polymer chains reach their fully extended state. In the limit case of *J*_*m*_ → ∞, the Gent model reduces to the neo-Hookean model (Eq. (2)). As *J*_*m*_ gets smaller, the inextensibility of polymer chains rigidifies the material at smaller strains. A comparison of the stress-strain relationship for the Gent model for a moderate value of *J*_*m*_ versus the neo-Hookean model is shown in Fig. 6. These hyperelastic (i. e. non-linear) models are contrasted with models of a linear stress-strain response.

### 4.3 Material anisotropy

There is an important distinction to make between material anisotropy and growth anisotropy. The former refers to elasticity while the latter is purely about geometry. Material anisotropy means that if one stretches the material in a certain direction, a certain force response will occur; but if the material is rotated, the measured force response might be different. For biological tissues, this is typically due to the presence of fibres in the material with a preferred orientation (Langer lines in skin are a familiar example). Unless such microstructural details are explicitly known, isotropic materials (in which the stress-strain behaviour is independent of material orientation) are usually preferred for modelling.

### 4.4 Growth anisotropy

**Fig. 7:**
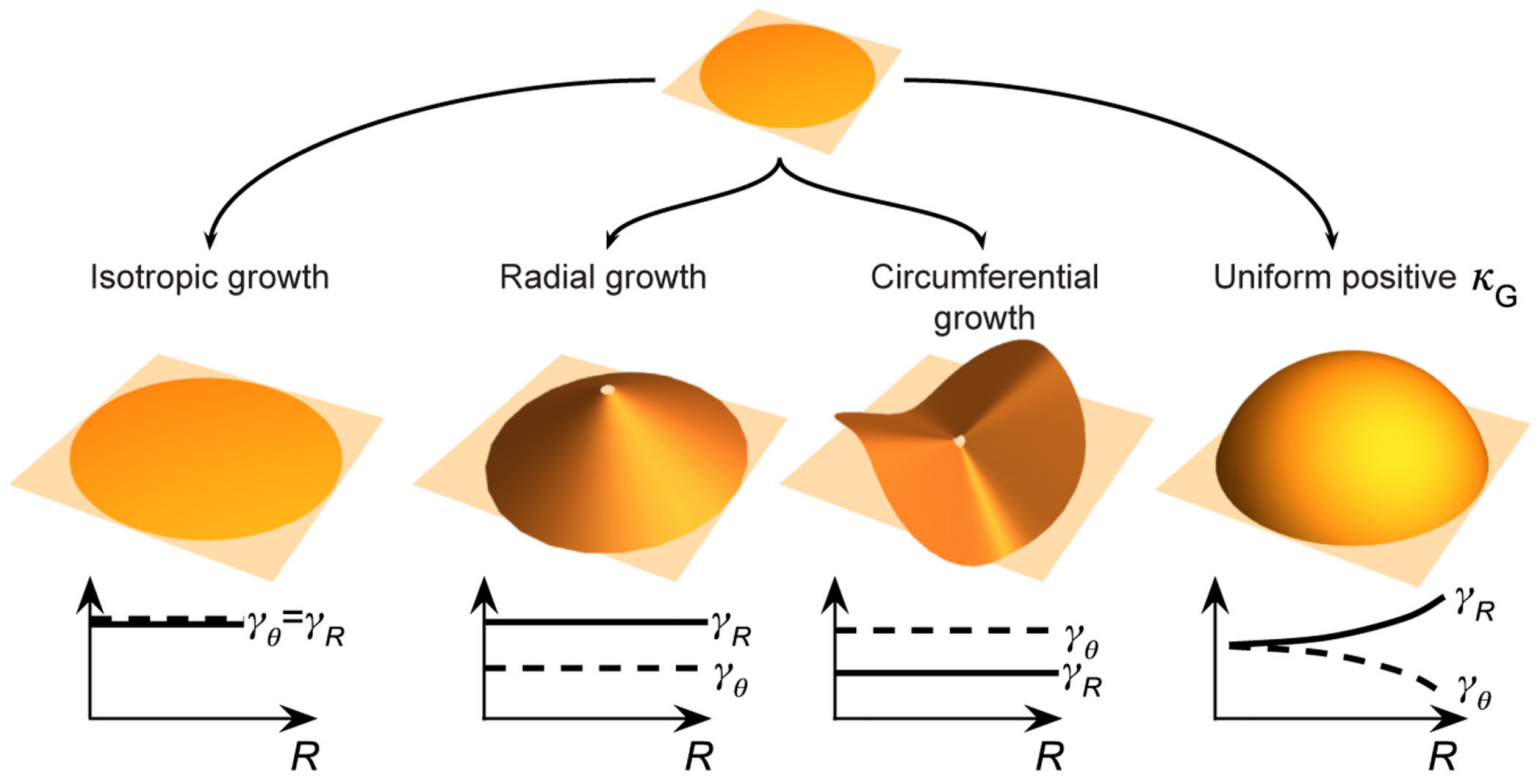
Stress-free reference configurations ℬ_*r*_ of grown 2D discs. These idealised shapes would be approached by real elastic sheets that are sufficiently flat, and when growth is sufficiently large to cause buckling. Growth anisotropy, described by differing growth factors in the radial (*γ*_*R*_) and circumferential (*γ*_*θ*_) directions, produces distinct stress-free shapes: isotropic growth (*γ*_*R*_ = *γ*_*θ*_) maintains planarity, radial growth (*γ*_*R*_ > *γ*_*θ*_) forms a cone, and circumferential growth (*γ*_*θ*_ > *γ*_*R*_) forms a saddle. Non-uniform and anisotropic growth patterns, such as growth under uniform positive Gaussian curvature *κ*_*G*_, can yield other complex shapes. Radial growth and circumferential growth surfaces adapted from [140].

An entirely separate mechanism from material anisotropy is growth anisotropy, meaning that during growth, material may be added differently in different directions of the tissue. To accommodate anisotropic growth, an elastic deformation partially absorbs these growth mismatches, and the material response itself can be either isotropic or anisotropic (e.g. if fibres are present). Let us consider a 2D disc. The growth tensor **F**_*g*_, written in an orthonormal polar basis, has the following components:

(4) $$F_{g}=\left(\begin{array}{cc} \gamma_{R} & 0\\ 0 & \gamma_{\theta } \end{array}\right)$$

here, *γ*_*R*_ and *γ*_*θ*_ are growth stretches, representing growth in the radial and circumferential directions. If their values are 1, then the multiplicative decomposition in Eq. (1) reads **F** = **F**_*e*_, and the morpho-elastic problem reduces to regular elasticity without growth. If, however, *γ*_*R*_ and *γ*_*θ*_ differ from 1, and/or differ from one another, and/or change throughout the tissue, various growth patterns emerge. For example, if we consider a flat 2D disc and add material in different patterns using different choices of *γ*_*R*_ and *γ*_*θ*_, we will obtain different stress-free configurations, i.e. the shapes that the disc would ideally assume if bending or stretching were not penalised by elastic energy. These are precisely the stress-free reference configurations ℬ_*r*_ (see Fig. 5). It is thus purely geometric and independent of the choice of an elastic material model. A selection of growth scenarios is illustrated in Fig. 7. If growth is isotropic (*γ*_*R*_ = *γ*_*θ*_) and spatially uniform, then growth is compatible, and the post-growth disc remains flat. In other cases, growth is incompatible, and the stress-free disc is non-planar. If more material is added radially than circumferentially (*γ*_*R*_ > *γ*_*θ*_), a cone shape results; if the opposite is true (*γ*_*θ*_ > *γ*_*R*_), a saddle shape appears. Many other patterns are possible if *γ*_*R*_ and *γ*_*θ*_ are spatially non-uniform. For instance, if the Gaussian curvature of a metric tensor constructed from **F**_*g*_ is positive and uniform, with *γ*_*R*_ and *γ*_*θ*_ non-uniform and anisotropic (*γ*_*R*_ ≠ *γ*_*θ*_), the resulting stress-free shape can resemble a spherical cap. This last scenario is identical to Fig. 4A.

## 5 Differential growth patterns that drive tissue-intrinsic stresses

### 5.1 Different growth strategies and their associated morphologies

We have seen a multitude of possible growth patterns, even for relatively simple systems like a growing disc. Based on the choice of growth patterns, the disc may relax into a cone-like, saddle-like, or hemisphere-like shape, all of which are *incompatible* growth patterns with their unique shape and residual stress profiles (shown in Fig. 7). Note that the shapes in Fig. 7 are idealised stress-free shapes in which sheets would fully relax if elasticity and other constraints were not a factor; real sheets would increasingly deviate from these shapes the thicker they are, or might not buckle at all if growth is not sufficiently large (see Appendix A). The large variety of shapes begs the question how among the plethora of possible growth patterns that lead to different morphologies Nature may select its growth patterns, i.e. what may be the particular growth strategies that result in observed patterns.

**Fig. 8:**
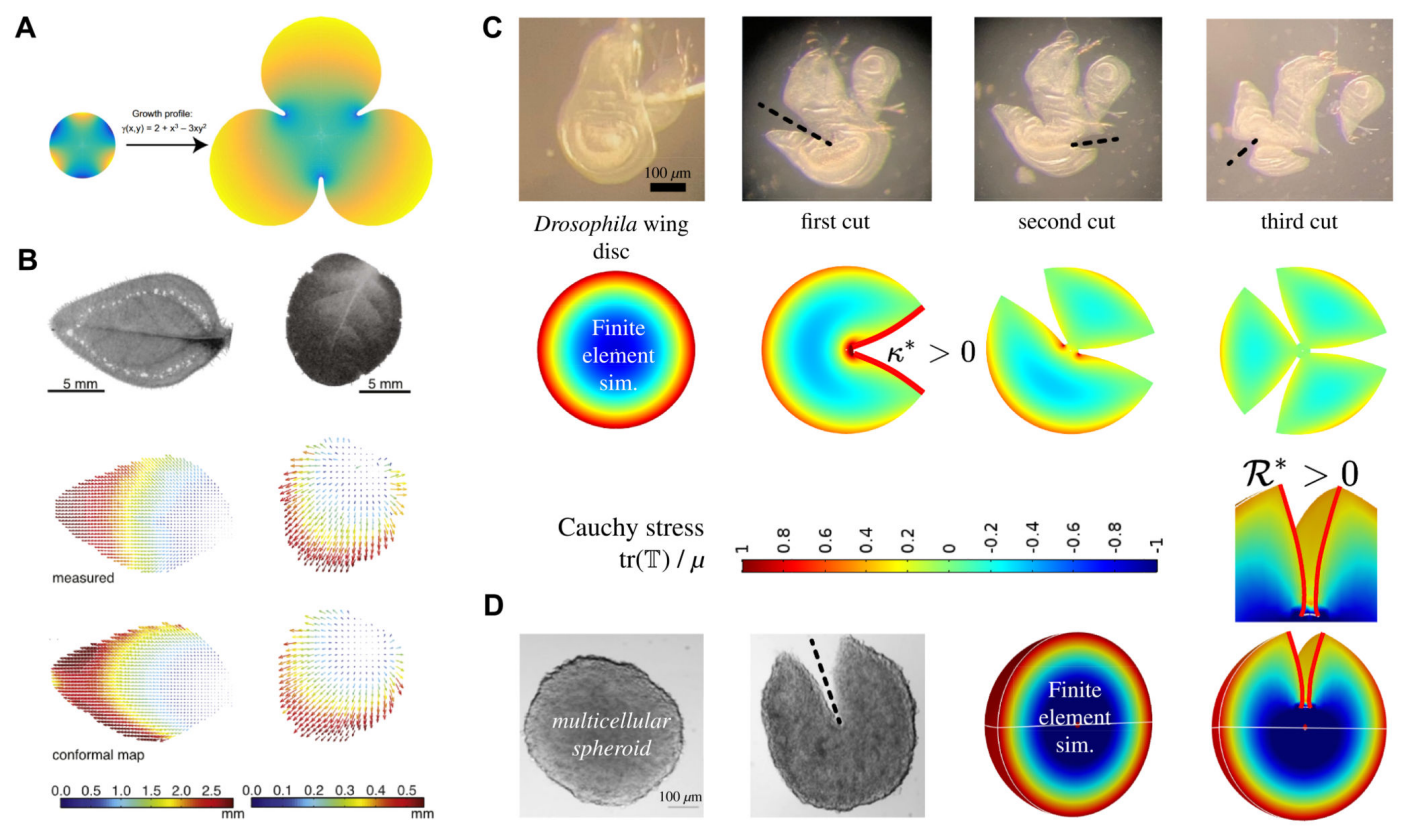
Different growth strategies. (A) “Harmonic growth” is a growth strategy in which the growth rate *γ* is a harmonic function, ∇^2^*γ* = 0. This mode of growth does not increase the residual stress in the tissue, even if prior to harmonic growth, some stress was accumulated. Figure adapted from [73]. (B) Harmonic growth patterns were experimentally observed in the growth dynamics of different leaves (*top*), *Petunia* (left column) and Tobacco (right column). The experimentally measured (*middle*) material displacement fields during leaf growth are very similar to the calculated displacement fields expected for a harmonic growth pattern (*bottom*). Panel originates from Ref. [5], licensed under CC BY 4.0. (C-D) Incompatibility-driven growth [45] explicitly accounts for the energetic cost of building tissue-internal frustration, approximated as Gaussian curvature in 2D (denoted κ* in the figure) and scalar Ricci curvature in 3D (ℛ* in figure). Uniform constant scalar curvature of the growth pattern qualitatively reproduces elastically relaxed opening patterns after single and multiple cuts (position of cuts are indicated by dashed lines) in different morphogenetic systems, the *Drosophila* wing disc (C) and multicellular spheroids (D). Panels C and D originate from Ref. [45], licensed under CC BY 4.0.

Two such growth strategies, that are mathematically closely related but follow distinctly different ideas, have been proposed recently under the name of *harmonic growth* [73] and *incompatibility-driven growth* [45]. Fig. 8 provides a visual representation of these growth strategies.

Harmonic growth is a smooth transformation that preserves angles, but not necessarily areas. It is characterised by a growth rate, *γ*, that satisfies the Laplace equation, ∇^2^*γ* = 0, indicating a harmonic function (see Fig. 8A). This condition ensures that the growth process does not introduce additional residual stress into the tissue, even if prior stresses existed, as shown in [73]. Harmonic (conformal) growth is isotropic and has been extended to an anisotropic setting via quasi-conformal maps, although so far with limited incorporation of mechanics [28]. This paradigm balances local expansions and contractions within the tissue, not to relax pre-existing stress, but to avoid additional stress. Experimental studies, such as those by Alim et al. [5], have demonstrated that leaf growth in *Petunia* and Tobacco closely follows harmonic patterns (Fig. 8). These findings suggest that harmonic growth may represent an optimal strategy for planar tissues, where stress avoidance is crucial for maintaining flat, undistorted morphologies.

In contrast to harmonic growth, incompatibility-driven growth explicitly incorporates the energetic costs of creating new incompatibility. In other words, growth is hypothesised to follow a pattern that doesn’t simply create an arbitrary incompatible pattern (e.g. any incompatible pattern from Fig. 7). Instead, a specific incompatible pattern is chosen in which the energetic cost of creating incompatibility, and the energetic cost of elastic deformation, are *minimal* in their sum total. As there is not one single way of quantifying incompatibility, but several, a choice is made to approximate incompatibility through Gaussian curvature of a thin sheet, or its full 3D generalisation, the Ricci scalar curvature (these concepts are elaborated in more detail in Appendix A). Gaussian curvature as a measure of incompatibility is closely related to the shape parameter of vertex models, a widely used cell-based model for epithelial morphogenesis [45].

As shown in Fig. 8C–D, uniform constant Gaussian curvature *κ*_*G*_ > 0 appears to effectively capture the stress distributions in various biological tissues. For instance, in the *Drosophila* wing disc and in multicellular spheroids, incompatibility-driven growth leads to residual stresses that manifest in characteristic opening patterns when tissues are cut. These mechanical responses align with predictions from finite element simulations, which model the growth tensor with prescribed curvature values.

While harmonic growth emphasises the avoidance of newly created stress, incompatibility-driven growth leverages controlled stress accumulation to regulate tissue size and shape. Together, these paradigms provide complementary perspectives on how biological systems achieve their diverse morphogenetic outcomes. The choice of growth strategy likely reflects the specific mechanical and functional demands of the tissue.

### 5.2 Growth-mechanical feedback loops

While the paradigms of harmonic and incompatibility-driven growth describe principles by which growth patterns may be selected, during morphogenesis growth patterns likely adapt in response to the local mechanical environment. To understand these dynamics, it is crucial to consider how growth-stress feedback loops influence and regulate morphogenesis. As growth advances, the residual stresses affect the growth pattern and force the tissue to adapt. Such growth-stress feedback loops are still in their infancy when it comes to understanding of the biochemical pathways.

Such dynamical adaptation of growth to stress is well-captured by the growth law:

(5) $${\dot{F}}_{g}F_{g}^{-1}=k^{+}\left(T^{*}-T\right),$$

where ${\dot{F}}_{g}F_{g}^{-1}$ describes the (potentially anisotropic) rate of growth, **T** is Eshelby stress, which is an energy-based measure of internal “driving forces” that result from changes in growth. Related is T*, the homeostatic stress tensor, which captures the baseline mechanical environment that maintains normal tissue structure and function. Here, *k*^+^ is a positive number that determines how quickly the Eshelby stress will converge to the homeostatic stress, if such convergence is possible. See Table 1 for an overview of the various stresses (*σ*, T, T*) and strains (**F, F**_*e*_, **F**_*g*_) that are of particular relevance to growth mechanics. In particular, the hypothesis behind mechanical homeostasis [8,42,111,82] is that at the homeostatic stress state **T** = T*, growth and shape changes do not occur $\left({\dot{F}}_{g}=0\right)$, as cellular processes of division, death and rearrangements balance each other out. Experimental evidence and physical understanding of homeostatic stress have been demonstrated in arteries, where residual stress was hypothesised by Y. C. Fung and others to homogenise the transmural stresses under physiological loading to minimise the tissue abrasion over the lifetime [27]. The latter has been captured with models of the type (5), which can lead to incompatibility that is consistent with small transmural stress gradients as well as post-cut opening angles consistent with experiments [132]. There is increasing experimental evidence that some living systems, such as embryos [15,16] and fibroblast cells [47], maintain such a target or homeostatic stress. The latter is likely evolving over the lifetime of an individual [46,128].

One central dogma in the context of developmental biology that has been clearly experimentally demonstrated is that tension induces cell proliferation, whereas compression inhibits it [63]. Mechanical homeostasis, exemplified by the growth law (5), provides a simple ansatz to capture this dogma. In a simplified setting, it can be written as:

(6) $$\gamma =k^{+}\left(\text{σ}-{\text{σ}}^{*}\right)$$

where *γ* is a growth rate, *σ* is a local value of stress, and σ* the value of homeostatic stress. Let us assume that the tissue’s preferred (homeostatic) state is in tension, σ* > 0. Then, if the tissue is locally in too much tension (*σ* > σ*), the tissue will grow (*γ* > 0) to relieve the tensile stress and approach its homeostatic value. Conversely, if locally the tissue is in too much compression σ < σ*, the tissue will resorb (*γ <* 0) to relieve the compression, which is a primitive but effective model for necrosis [44].

**Fig. 9:**
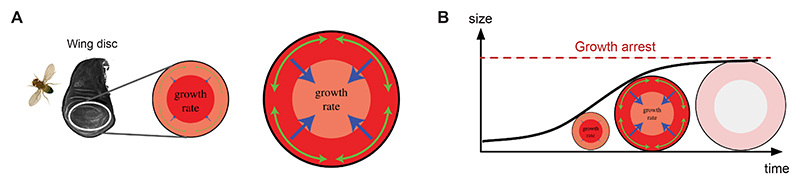
Feedback loops between growth and stress. (A) The *Drosophila* wing disc is an epithelial tissue that grows in the *Drosophila* larval and forms the fly wing during metamorphosis. The growth rate is indicated by different intensities of red (high intensity = fast growth). Differential growth rates, fast in the centre and slower in the periphery, have been proposed to lead to stress-accumulation during disc growth: The initially faster growing central cells push outwards and stretch the peripheral tissue (green arrows). The slower growing peripheral tissue in turn constraints central growth and leads to compression of the central domain (blue arrow). (B) Once the level of compression in the centre exceeds a critical threshold, growth is hypothesised to stop, with the tissue assuming its final size. Figure adapted from [1].

A feedback loop comparable with (5) coupling tissue growth and stress in such a way was used by Aegerter Wilmsen and co-workers [1] to offer a possible explanation for how size is regulated in the *Drosophila* wing disc (see Fig. 9). In the wing disc, most growth is believed to initially occur at the disc centre causing lateral tissue to get stretched (green arrow in Fig. 9A) and the centre consequently to be compressed (blue arrows). Growth terminates, and a final size is obtained, once a sufficient level of compression is reached (Fig. 9B). The mechanism relies crucially on features such as thresholding and morphogen diffusion which we omitted here for clarity. While such a hypothetical purely 2D explanation of size control of the specific system of *Drosophila* is weakened in light of recent findings showing the importance of 3D shape [59, 139], it provides nevertheless an insightful guiding hypothesis of stress-influenced morphogenesis (independently, [70] developed a similar hypothesis almost simultaneously).

### 5.3 Mixture theories

The previous section considers a solid, elastic material. However, many applications require the coexistence of multiple materials, for example a solid skeleton interpenetrated by fluid, or an elastic material made up of several intermingled components (e.g. elastin and collagen). Mixture (multiphasic) theories start from the observation that tissues contain co-located ingredients, such as cells, matrix, and water. Each has its own mechanics and turnover. Instead of one homogenized continuum, they describe several interacting phases that exchange mass and momentum in the same region.

Broadly, mixture approaches can be divided into two categories. *Solid*–*fluid mixtures* consider a porous solid scaffold (e.g. cells and ECM) which is permeated by interstitial water. Classical poroelasticity [30], equipped with a multiplicative decomposition for the solid phase, has been applied to proliferating spheroids [151] and plant growth [101]. A separate approach are *Solid*–*solid mixtures*. Several solid constituents deform together but can grow or remodel at different rates. Constrained mixture theory assigns each phase its own mass production and removal laws while averaging their stresses [11,71]. Dynamical-systems extensions introduce feedback rules that predict whether coupled turnover drives tissues toward a stable homeostatic size or pathological enlargement (e.g. aneurysms) [33,82]. Constrained mixture models have been integrated with intracellular signalling and chemo-mechanical coupling [105]. These models, while computationally complex, have had success especially in cardiovascular modelling.

## 6 Residual stress shapes growing multi-layer structures

In the previous section, we saw how non-uniform growth within a single tissue can generate internal stresses. In reality, tissues rarely expand freely; they encounter neighbouring tissues, rigid structures, or the extracellular matrix (ECM). Whenever a growing region is constrained by another structure—whether stiffer, slower-growing, or both—this mismatch can produce residual stresses and, over time, mechanical instabilities such as buckling. Thus, even uniform expansion can accumulate stress if it meets external resistance.

A tissue’s mechanical environment is dynamic: surrounding structures also grow, remodel, or adjust their stiffness, thereby shaping—and being shaped by—the expanding tissue. In some cases, the environment imposes passive obstacles; in others, the tissue actively modifies its surroundings, for example by remodelling the ECM. Below, we illustrate these ideas through two main scenarios: the interplay between different tissue layers (“tissue-tissue”) and interactions with the ECM (“tissue-ECM”).

### 6.1 Differential growth between connected multilayered tissues

Examples of unconstrained tissue growth are relatively rare, as growing tissues are typically embedded within a developing organism and subjected to complex boundary conditions. In addition, organs often exhibit intricate, multilayered structures that support their specialized and diverse functions. These layered configurations also play a crucial role in distributing mechanical stresses and accommodating growth-induced deformations, contributing to the resilience and stability of organs such as arteries [76]. Organ growth and morphogenesis depend on the synchronized expansion and shape transformation of multiple tissue layers to ensure cohesive development and structural integrity. However, differences in growth rates between layers within a multi-layered structure can create geometric incompatibilities and internal stresses. Nature often resolves these mismatches by allowing tissues to bend, wrinkle, or curve, thus shaping complex organ morphologies.

**Fig. 10:**
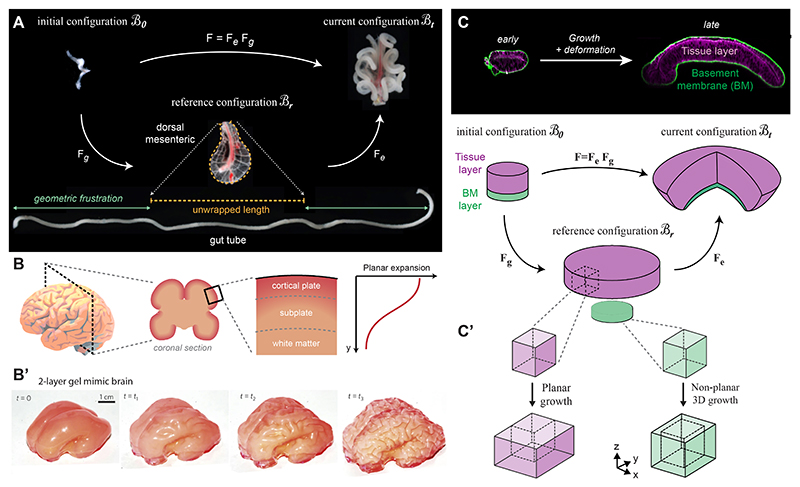
Differential growth between coupled multi-layers. (A) Multiplicative decomposition of chick gut morphogenesis. During development, the gut tube grows predominantly along its principal axis compared to the dorsal mesenteric, leading to a geometric incompatibility (revealed by the separation of the two tissues in the *reference configuration* ℬ_*r*_). This incompatibility leads to elastic deformation (compression of the gut tube and stretcohing of the dorsal mesentery) and residual stress, driving the stereotypic looping pattern of the grown gut (in the current configuration ℬ_*t*_). (B) Differential expansion of the cortical plate (*planar*) versus the inner white matter (*non-planar*) mechanically drives the folding pattern of the cortex. This mechanism can be illustrated by using a two-layer gel model, where the peripheral gel layer swells more (B’), leading to folding patterns that resemble human cortex morphology. (C) *top*: Section views of *Drosophila* wing disc epithelium (purple) covered by a Collagen IV-rich BM (green). *middle*: Multiplicative decomposition of the tissue (purple) and BM layers (green). Differential growth anisotropy between the tissue (planar) and the BM (non-planar, see C’) lead to an incompatibility, i.e. two discs of different size in the reference configuration. Elastic deformation, at the cost of residual stress, connects the two discs in one coherent but bent object. (a) Panel A is adapted from [114]. (b) Panel B is adapted from [134]. (c) Panel C is adapted from Ref. [59], licensed under CC BY 4.0.

The looping of the vertebrate gut is an illustrative example of how differential growth between two layers leads to internal stress that instructs functional morphology on the organ level. The chick gut tube forms highly stereotypic loops in order to fit the grown gut in the body cavity. Savin et al. showed that this looping pattern is the result of differential growth between the gut tube and the dorsal mesentery which the gut tube is anchored to [114]. The faster growth of the gut tube compared to the slower growing dorsal mesenteric leads to an incompatibility and internal stress that is driving the looping patterns of the chick gut (see Fig. 10). Notably, internal stress and the incompatibility can be visualized when the fully looped gut tube is separated from the dorsal mesenteric by surgical separation, thereby visualising the reference configuration ℬ_*r*_ (Fig. 10A): Upon uncoupling the two tissue layers, the gut tube expands and relaxes into a linear unbent geometry, that is geometrically incompatible with the much smaller dorsal mesenteric (see dashed orange line in Fig. 10A). This relaxation behaviour indicates that the looped gut tube (and the dorsal mesenteric) must have been internally stressed and deformed in the current configuration ℬ_*t*_. Importantly, the build-up of internal stress and the resulting deformation (looping) depend on the two tissues to be mechanically linked, as the surgical removal of the dorsal mesenteric early during development disrupts the formation of gut loops (see [114]). Using a computational model, the authors further validate that the geometry and mechanical properties of the tissues dictate the buckling wavelength and pattern and the model accounted for distinct looping patterns observed in other bird species and in mouse.

A recent study from the Mahadevan lab established a data-informed multilayer model to further explore how differential mechanical properties along the chick gut contribute to the formation of distinct buckling patterns in the oesophagus, small intestine, and large intestine [50]. Using the concept of morphoelasticity and informed by experimentally determined geometries, material properties, and growth rates, the researchers demonstrate that variations in these parameters lead to region-specific mucosal folding through primary and secondary buckling mechanisms. Consistently, mechanically enforcing cell crowding and multilayering leads to a mechanical instability that enhances gut spheroid formation [86].

The folding patterns of the cerebral cortex have also been attributed to differential expansion of the outer cortical layer versus the inner core (white matter, see Fig. 10B’) [14,133]. This mechanism was illustrated using 3D-printed layered gel mimics of the brain [134], where the increased swelling of the outer layer (representing planar cortical growth) relative to the core drives compression of the outer layer and a folding pattern that resembles the one observed in the fetal brain (see Fig. 10B–C). Recent studies have further explored this mechanism, showing that faster growth of the outer layer creates an outward (radial) force, while the slower growth of the core acts as geometric constraint, leading to circumferential tension in the core [83]. Furthermore, mechanical modelling has explored how local variations in stiffness- and growth-ratio and the initial cortical thickness impact the normal folding pattern and how variations of these three parameters can lead to cortical malformations [145].

Differential growth between physically linked tissue layers, and the resultant stresses have been implicated as morphogenetic mechanism in several other processes, including the folding pattern of the surface of our intestine [17,120], during heart tube [119] and foregut formation [69] and during early tooth morphogenesis [89]. In all these examples, spatial differences in either the rate or the anisotropy of growth (or a combination of both) lead to incompatibilities that provoke the slower-growing parts to act as a geometric constraint limiting the expansion of faster-growing parts. As a consequence, such growth patterns lead to the accumulation of internal stresses (as observed by tissue cuts, see Fig. 8C–D and Fig. 10A) and provide forces for tissue-scale deformations. Therefore, these examples illustrate the role of stresses generated by growth differences between physically connected tissue layers in guiding functional organ morphology during development.

### 6.2 Differential growth between tissues and their ECM

While the previous section has focused on how differences in tissue growth lead to internal stresses, incompatibilities and growth-induced stresses can also arise from mechanical interactions between growing tissues and their extracellular environment. The extracellular environment is primarily represented by the extracellular matrix (ECM), a complex network of proteins and polysaccharides that provides structural and biochemical support to cells [143]. The ECM not only serves as a physical scaffold but also influences cellular behaviours such as migration, differentiation, and proliferation through biochemical signalling [113,149].

Epithelial tissues are lined by a specialized sheet-like ECM known as the basement membrane (BM). Traditionally, the BM was viewed as a passive, rigid boundary that constrained tissue expansion during morphogenesis. However, it is now understood to be a highly dynamic structure [36,123]. Recent findings show that the BM undergoes continuous remodelling, actively contributing to morphogenesis by altering tissue shape and mechanics. This remodelling is regulated by cellular processes, including the secretion of matrix metalloproteinases, which degrade BM components, and the rearrangement or deposition of BM structural proteins [80,81]. These processes modify the BM’s mechanical properties, such as stiffness and elasticity, in response to developmental cues [137].

Importantly, the BM functions as a dynamic boundary condition for growing tissues. Its mechanical properties feed back on tissue shape by influencing stress distribution and guiding tissue deformations. There are increasing examples of the extracellular environment acting as geometric constraint for growing tissues, hindering their expansion and leading to tissue deformation. Examples of the mechanical interplay of tissue growth and dynamic remodelling of the surrounding BM include tissue folding [59,102,125,126,139], anisotropic expansion [32,57,81, 138], and branching [61,95,144] (please refer to an excellent recent review by the Stramer lab for more examples [36]). In addition, theoretical studies and mechanical simulations have started to provide conceptual understanding of the mechanical interplay between tissue growth and BMs to determine morphology [7,22,148].

While these examples emphasise the active role of BMs in mechanically shaping tissues during morphogenesis, our mechanistic understanding of how BMs influence the morphogenetic forces and stresses that drive organ shape remains limited. Importantly, BMs are associated with the accumulation of internal stresses during organ growth, as revealed by tissue relaxation upon enzymatic digestion of the collagen IV-rich BM layer by Collagenase in the *Drosophila* wing disc [59,94,104] or the vertebrate optic cup [102]. These findings demonstrate that insufficient matrix expansion can hinder epithelial growth, leading to accumulation of internal stress and tissue deformation. We recently examined how anisotropic (directionally variable) growth of the BM relative to the epithelial cell layer leads to the accumulation of internal stress that drives 3D shape formation in the *Drosophila* wing disc [59]. The wing disc epithelium is encapsulated by a collagen IV-rich BM. During wing disc growth, the initially flat disc bends to form a dome-like structure (see Fig. 10C). This bending is due to the BM lining the basal side of the wing disc, which grows in a different pattern than the epithelial tissue: The epithelial tissue expands by planar growth, i.e. it expands predominantly along the horizontal axis with no major active change in tissue height (see Fig. 10C’ *left*). In contrast, the underlying BM expands by a non-planar growth pattern that leads to an increase in BM height, but also to less expansion along the horizontal axis. This differential growth anisotropy causes a geometric mismatch, i.e. the two discs have grown to different sizes (see Fig. 10C reference configuration). As in previous examples, compatibility between the two discs is restored by introducing residual stress and elastic deformation, helping the tissue develop its complex bent 3D morphology. Notably, cellular control over the anisotropy of BM growth involves Matrix Metalloproteinase 2 (MMP2), an enzyme well known Collagen IV remodelling. In the wing disc low epithelial MMP2 expression is associated with non-planar BM growth (leading to compressive stress in the tissue), while moderate expression of MMP2 allows planar BM expansion (and reduced tissue compression). Therefore, MMP2 provides a genetic link between developmental patterning and the mechanical environment of a growing tissue, thereby tuning external constraints, stresses and tissue deformation. Future studies will provide a more detailed understanding of the cellular capacity and regulatory mechanisms that endow growing tissues to instruct BM mechanics and growth to accommodate tissue expansion during morphogenesis.

Notably, major morphogenetic shape changes during normal animal development are also associated with local protease-mediated degradation of BMs, such as the elongation of the *Drosophila* pupal wing and leg disc [34,35]. While the role of any accumulated internal stresses has not been tested during *Drosophila* pupal development, it is tempting to speculate if developmentally accumulated internal stresses and their acute relaxation assist in fuelling fast shape changes during metamorphosis.

Collectively, these studies support a key role for the dynamic regulation of the ECM’s mechanical properties, including material properties such as stiffness but also its growth anisotropy, in mechanically determining final organ shape. ECM mechanics is a central aspect of morphogenesis, and points to the ECM not just as a passive scaffold but as a dynamic participant in developmental shape generation, with growth anisotropy playing a decisive role in morphogenetic processes. Furthermore, these findings underscore that growth differentials and geometric boundaries are universal principles governing tissue shape during morphogenesis.

## 7 Conclusion

Unravelling how growth-induced stresses influence tissue shape provides a richer, more integrated perspective of animal morphogenesis. While myosin-based contractility has long taken centre stage in shaping developing tissues, the examples and theoretical frameworks presented here highlight that growth can be an equally potent driver of morphological transformations. By considering growth as an active process capable of generating internal stresses, we gain new insights into how cells navigate mechanical constraints, remodel their extracellular environment, and collectively orchestrate complex three-dimensional forms. At the same time, geometric constraints, once largely neglected, are now recognized as playing a critical instructive role: distinct boundaries, such as stiffer neighbouring tissues or elastic basement membranes, can bias the direction of tissue expansion, thereby guiding folds, bends, and curvatures. These constraints ensure that growth does not simply scale organs uniformly, but instead helps them assume functional, context-specific shapes. In this way, geometric boundaries act not merely as passive obstacles, but as active participants in defining the spatial patterning of shape changes during animal development.

Importantly, stresses arising from growth are rarely isolated phenomena; they emerge at the interface of multiple processes. Spatially varying growth rates, differential expansion between tissue layers, or mismatches with extracellular matrices can all generate elastic instabilities. These instabilities, in turn, can guide folding, bending, and buckling events that help sculpt organs ranging from the intestine to the brain. The interplay of growth, mechanical feedback, and ECM remodelling underscores that morphogenesis is not only a genetic and biochemical process but also a physical one, where geometry and elasticity must be considered integral players. In this context, discussing differences in tissue growth and the more recent observation of tissue/BM growth is essential to understand how active remodelling of the extracellular environment can shape developmental trajectories.

A promising avenue of future research lies in integrating quantitative mechanical measurements with advanced imaging and computational modelling. Such a synthesis can help bridge the knowledge gap, providing a clearer understanding of the relative importance of growth-driven stresses compared to other mechanical or chemical cues. By developing predictive theoretical tools and validating them through experiments, we may better grasp how tissues achieve their final sizes and shapes. The recent inflow of physical and computational tools has transformed the field of morphogenesis and led to unprecedented insights into the mechanical logic of shape generation. In this review we tried to highlight the insight that can be gained by data-informed modelling frameworks and it will be exciting to see future advances in the field of morphogenesis; well in line with the vision of D’Arcy Wentworth Thompson, who more than 100 years ago correctly stated: “I believe the day must come when the biologist will, without being a mathematician, not hesitate to use mathematical analysis when he requires it.” [12].

## Supplementary Material

Appendix

## Figures and Tables

**Table 1:** Glossary. Next to the symbol, units are given in angular brackets.

Symbol	Definition
**F** [1]	**Total deformation gradient**: Describes how each portion of a tissue moves and distorts from its original initial (pre-grown) configuration to its actually observed shape. Includes rotation and stretch.
**F***_g_* [1]	**Growth tensor**: Accounts for the permanent, biologically driven changes in tissue size and shape (e.g., growth or remodelling).
**F*_e_*** [1]	**Elastic deformation gradient**: Captures the purely elastic, reversible part of the tissue’s deformation in response to forces.
*μ* [Pa]	**Shear modulus**: Quantifies the tissue’s resistance to shape changes (shearing) and is related to Young’s modulus, which quantifies resistance to extension. *μ* equals the small-strain shear modulus; at large strain its value depends on the chosen stress-strain relationship.
*σ* [Pa]	**Cauchy stress tensor**: Describes the actual stresses within the tissue in its current (deformed) configuration.
**T** [Pa]	**Eshelby stress tensor**: An energy-derived measure that represents how internal material rearrangements, such as growth and incompatibility, generate driving forces within a tissue.
T* [Pa]	**Homeostatic stress tensor**: The homeostatic stress tensor is the stress state that a living tissue tends to maintain or restore under growth and remodelling, representing its preferred mechanical state. **Residual stress**: The stress that remains in a tissue even when all tractions and body forces have been removed.
