# Supplementary material for: How growth-induced stresses guide shape changes during animal morphogenesis: Mechanisms and implications
Source: Semin Cell Dev Biol. Author manuscript; Available in PMC 2026 Mar 15. (PMC7618882; doi:10.1016/j.semcdb.2025.103661)
Supplement: Appendix [file EMS212899-supplement-Appendix.pdf]

Appendix A

Gaussian and scalar Ricci curvature

When a tissue grows, the distances that separate neighbouring cells change. A convenient way to describe these local changes is to imagine that the tissue is constantly rewriting its own "internal ruler". Mathematically, that ruler is summarised by the growth tensor $\mathbf{F_g}$. If two material points $\mathscr{B}_r$ were

originally separated by the infinitesimal distance d$\mathbf{X}$, after growth the separation they would like to have (before any elastic forces act) is $d\ell^2 = d\mathbf{X}^T\mathbf{F}_g^T \mathbf{F}_g\, d\mathbf{X}$, where $\mathbf{F}_g^T\,\mathbf{F}_g$ plays the role of the new, growth-adjusted notion of distance (a metric tensor). Whenever different regions choose different rulers, the sheet or bulk of tissue cannot lie comfortably in its old (pregrowth) shape; it must either stretch or bend to accommodate the incompatibility.

Gaussian curvature – the 2-D case

For a thin epithelial layer, we can treat the tissue as a two-dimensional surface. The single number that matters is the Gaussian curvature κ_G. Positive κ_G means the surface would like to relax into a dome-like shape, negative κ_G that it prefers a saddle, and $\kappa_G = 0$ the relaxed state is flat (at least locally). When growth stretches the tissue differently in the radial (γ_R) and circumferential (γ_θ) directions of a disc, κ_G is given by:

$$\kappa = \frac{(R\gamma'_\theta + \gamma_\theta)\gamma'_R - R\gamma_R\gamma''_\theta - 2\gamma_R\gamma'_\theta}{R\gamma_\theta\gamma_R^3} \tag{A.1}$$

where the prime denotes a derivative with respect to the reference coordinate R in $\mathscr{B}_0$ [45,140]. Let us assume, as a simplifying case, that γ_R and γ_θ are constants (not dependent on R). This corresponds to the first three scenarios shown in Fig. 7. If $\gamma_R = \gamma_\theta$ then the curvature (A.1) vanishes and no bending is required, i.e. a flat disc remains a flat disc. If, on the other hand, $\gamma_\theta > \gamma_R$, then the curvature κ_G becomes negative and a saddle forms; if $\gamma_R > \gamma_\theta$ then κ_G is positive and a cone develops. Trying to keep such a disc perfectly flat, as in an in vitro experiment constrained between glass slides, would create residual stress in the flattened disc as it needs to stretch locally due to the incompatibility.

Scalar Ricci curvature – the 3-D case. Inside a thicker organ there is a third dimension, and one scalar is no longer enough to fully characterise intrinsic curvature. Instead, we use the Ricci tensor R_{AB}. Its formal definition involves the full Riemann curvature [26]. Adding up the diagonal entries of R_{AB} gives the scalar curvature $\mathcal{R}$; in a thin sheet that scalar reduces to $2\kappa_G$, so the 2-D discussion is contained as a special case.

Usually, the Ricci curvature scalar $\mathcal{R}$ or the Gaussian curvature κ_G is imposed in problems of morphoelasticity, or instead is accumulated indirectly from evolution equations based on stress or chemical gradients. However, recent morphoelastic models [46,45] consider a direct energy penalty related to curvature $\mathcal{R}$ (or in 2D κ_G). The energetic penalty of curvature models the creation of material defects (incompatibility).

Summary. Intrinsic curvature acts as a "geometric seed" for residual stress. If one measures or infers how much growth stretches one direction relative to another (i.e. finding $\gamma_R(R)$, $\gamma_\theta(R)$), and convert those stretches into κ for a layer (via Eq. (A.1)) or $\mathcal{R}$ for a bulk, one immediately knows (i) whether a sheet without any constraints would be planar, dome, saddle, or something more complicated, and (ii) where residual stresses must build up. In this way Gaussian and Ricci curvature create residual stress from the geometry of differential growth.
